# Revisiting the Maxillary Teeth in 384 Subjects Reveals A Deviation From the Classical Aesthetic Dimensions

**DOI:** 10.1038/s41598-018-36770-w

**Published:** 2019-01-24

**Authors:** María Melo, Fadi Ata-Ali, Julia Huertas, Teresa Cobo, Jamil Awad Shibli, Pablo Galindo-Moreno, Javier Ata-Ali

**Affiliations:** 1 Valencia University Medical and Dental School, University of Valencia, Valencia, Spain; 2 Universidad Europea de Valencia, Faculty of Health Sciences, Department of Dentistry, Valencia, Spain; 3 Department of Surgery and Medical-Surgical Specialities, Area of Orthodontics, University Medical and Dental School, University of Oviedo (Spain), Instituto Asturiano de Odontologia, Oviedo, Spain; 4 Department of Periodontology and Oral Implantology, Dental Research Division, Guarulhos University, Guarulhos, São Paulo Brazil; 5 Department of Oral Surgery and Implant Dentistry, School of Dentistry, University of Granada, Granada, Spain; 6 Public Dental Health Service, Conselleria de Sanitat Universal i Salut Pública, Generalitat Valenciana, Valencia, Spain

## Abstract

Dental esthetics need to be defined from the scientific perspective in order to obtain predictable treatment outcomes and avoid the effects of subjectivisms on the part of the dental profesional. It can be deduced that the ideal dimensions in the maxillary anterior sector are difficult to establish. Therefore, the primary purpose of this study was to define the dimensions of the maxillary anterior teeth and the relationships between them. In addition, an analysis was made to reinvestigate whether they complied with the Golden proportion, the RED (Recurrent Esthetic Dental) proportion and the Golden percentage. A total of 2304 tooth corresponding to 384 subjects were evaluated. The central incisor presented a mean width of 8.58 mm and a height of 9.35 mm, while the lateral incisor presented a width of 6.69 mm and a height of 7.75 mm. The mean width of the canine was 7.69 mm, with a height of 8.68 mm. The teeth revisited in this study did not comply with the ideal dimensions in the anterior maxillary sector as established by the Golden proportion, Golden percentage and RED. The information obtained from this study can be clinically applied to restore the dimensions during periodontal surgery, restorative dentistry and prosthetic rehabilitation.

## Introduction

Dental esthetics need to be defined from the scientific perspective in order to obtain predictable treatment outcomes and avoid the effects of subjectivisms on the part of the dental professional^1^. A series of reference patterns have been proposed in order to secure ideal outcomes, defining aspects such as upper lip contour, proximal contact points and anterior tooth proportions, among other variables^2,3^. Attempts have also been made to relate facial esthetics to dental esthetics through parameters such as interpupil and intercanine distance, nasal interalar width and mesiodistal distance of the maxillary anterior teeth^4,5^, inter-zygomatic width or width of the central incisor^5,6^.

In 1978, Levin proposed the theory of the Golden proportion^7^. It was postulated that the width of the upper lateral incisor, seen in frontal view, must be in golden proportion with the width of the central incisor. Another theory is that of the Golden percentage, proposed by Snow in 1999^8^. This investigator concluded that dividing the relative width of each tooth by the sum of the widths of all the teeth yields the percentage width occupied by each individual tooth in the intercanine distance seen in frontal view. On the other hand, Ward (2000) described the Recurring Esthetic Dental (RED) proportion^6,9^. In the case of the upper central incisors, a RED proportion of 70% is recommended for teeth of normal heigth, with a height/width ratio of 78–80%. If the anterior teeth are very short, the use of a RED proportion of 80% is more suitable, while in the case of long teeth a RED proportion of 62% is preferred, coinciding with the Golden proportion described by Levin^7,9^. The longer the teeth, the lower the recommended RED proportion^8,10^.

Based on the above, it can be deduced that the ideal dimensions in the maxillary anterior sector are difficult to establish, due to the great variability among subjects. Nevertheless, dental esthetics must be regulated by anatomical rules. Therefore, the primary purpose of this study was to define the dimensions of the maxillary anterior teeth and the relationships between them. On the other hand, an analysis was made to reinvestigate whether they complied with the Golden proportion, the RED proportion and the Golden percentage, in a sample of subjects with permanent teeth without prostheses or dental implants.

## Material and Methods

Plaster casts (upper and lower) of 384 subjects between 14 and 35 years old with a mean age of 20.1 years, obtained in the University of Valencia Dental School (Valencia, Spain) were evaluated (at 14 years old, the person has all the permanent teeth, except maybe the third molar that is not included in this study). The included subjects were of Spanish origin, with complete and fully erupted permanent dentition, and with no current or past orthodontic treatments. Subjects were excluded if they presented history of dentoalveolar trauma, incisal marginal wear, caries with the loss of tooth material, prosthetic reconstructions, anterior sector prostheses or dental implants, and periodontal disease. In addition, the included cases presented no negative or positive bone-dental discrepancies of the anterior teeth, giroversions, intrusions or extrusions, malformations or developmental anomalies or agenesis in the anterosuperior sector. Irreversible hydrocolloid imprints (Orthoprint, Zhermack, Badia Polesine (RO), Italy) were obtained of both maxillae, followed by the preparation of type II plaster casts (Snow White Plaster, Kerr, Bioggio, Switzerland). After removing the impression from the mouth of the patient, it was washed under running water, submerged for one minute in disinfectant solution, and then washed profusely. Casting was performed immediately. Written informed consent was obtained from all the subjects. In the case where participants are under age 18, written informed consent was obtained from each patient’s parent or legal guardian. The study was conducted in accordance with the Declaration of Helsinki, and was approved by the Institutional Review Board of the University of Valencia (Reference: H1476907919057).

**Figure 1:**
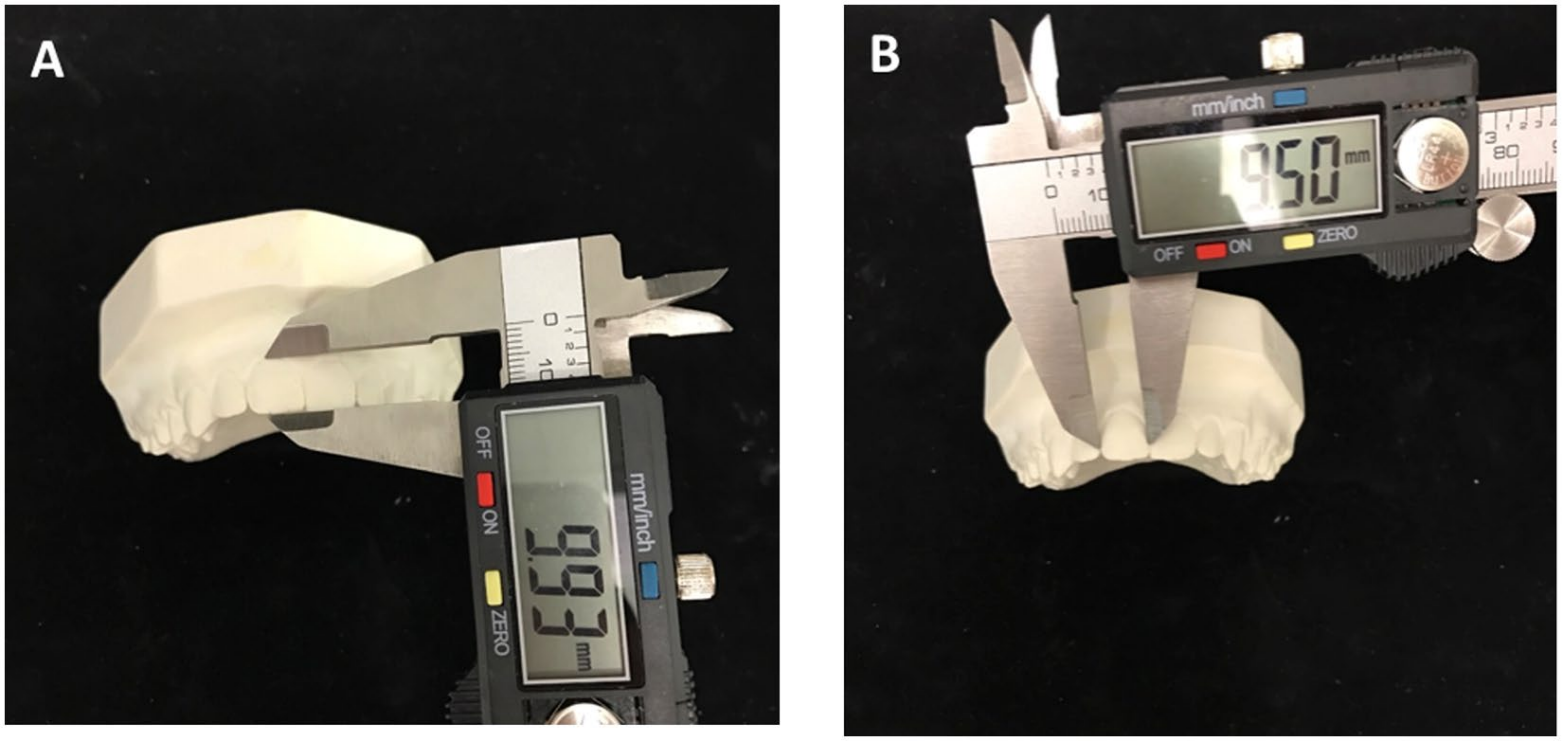
(**A**) Example of the measurement of height. (**B**) Example of the measurement of width.

Two trained investigators (JH and MM) recorded all the parameters. Two measurements were made and compared in each case in order to ensure reliability; if they differed by more than 0.2 mm the procedure was repeated, and if they differed by less than 0.2 mm the weighted average was calculated^11,12^. We first measured the heigth and width of the crowns of the 6 maxillary anterior teeth using digital calipers (Kreator KRT705004, Belgium) with a precision of 0.1 mm. The heigth of the clinical crown was measured from the gingival zenith to the incisal margin or cuspid tip along the longitudinal axis of the tooth. Width in turn was measured as the maximum distance between the mesial and distal contact points along a line perpendicular to the longitudinal axis of the tooth (Fig. 1). All measurements were recorded on the buccal surface of the teeth.

**Figure 2:**
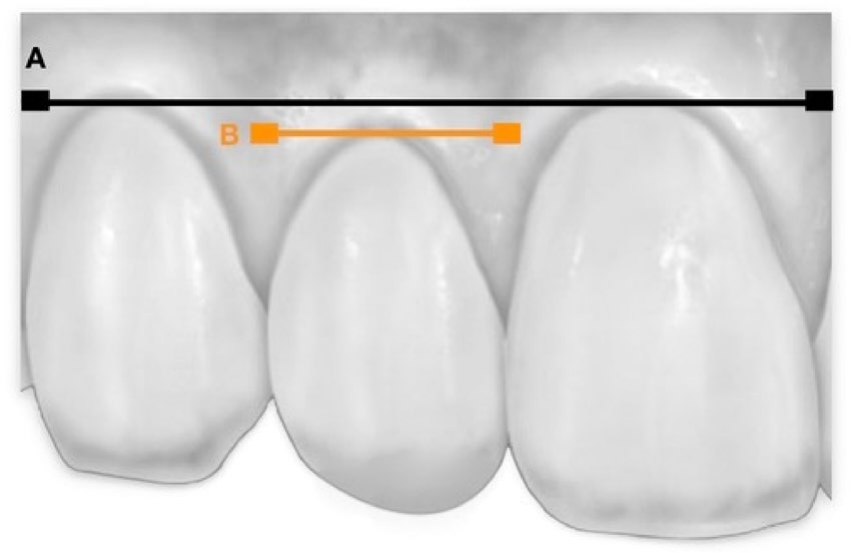
Line (**A**) joins the gingival zeniths of the central incisor and canine. Line (**B**) marks the gingival zenith of the lateral incisor. In this case, line B was located below line (**A**) and so is marked as negative.

In order to obtain the apico-coronal distance of the gingival margin of the lateral incisor (LI) with respect to the central incisor (CI) and canine (C), a pencil was used to mark the gingival zeniths corresponding to the central incisor, lateral incisor and canine on the plaster cast (Fig. 2). We then traced a perpendicular gingival line joining the gingival zeniths of the central incisor and canine. Finally, we measured the apico-coronal distance between the gingival zenith of the lateral incisor and the traced gingival line. If the gingival margin of the lateral incisor was located above this line, the value was marked as positive, while if the margin was located below the line it was marked as negative^13^.

In order to measure the apparent widths of the maxillary anterior teeth, photographs were obtained of all the casts in frontal view, based on a standardized protocol. All photographs were made by the same investigator (JH), using a digital camera (Canon EOS 77D, Tokyo, Japan), and were filed in JPG format for analysis. A calibrated digital ruler was used to measure apparent width (AW)(in mm). The corresponding LI/CI and C/LI ratios were calculated.

The following definitions were used in the present study:

- **Golden proportion:** This parameter states that the width of the upper lateral incisor, in frontal view, should be in Golden proportion with the width of the central incisor, i.e., the width of the upper lateral incisor should be 62% of the width of the central incisor, and the width of the canine should be 62% of the width of the lateral incisor. In other words, the width of the central incisor multiplied by the value defined as the golden proportion (0.618) yields the width of the lateral incisor, and the width of the lateral incisor multiplied by 0.618 in turn yields the width of the canine.
- **Golden percentage:** This parameter states that dividing the relative width of each tooth by the sum of the widths of all the teeth yields the percentage width occupied by each individual tooth in the intercanine distance seen in frontal view. The width of the upper central incisor should be 25% of the total width of the intercanine distance. Each maxillary lateral incisor should be 15% and each canine 10%, i.e., the proportional width of each tooth should be: canine 10%, lateral 15%, central 25%, central 25%, lateral 15% and canine 10%^8^.
- **Recurring esthetic dental (RED) proportion**: This parameter is based on a progression where the width of each successive tooth decreases in the same proportion, progressing distally. The true measures of the teeth are obtained from plaster casts. However, the apparent widths must be recorded from photographs, because they show us the patient in “frontal view”. In contrast to the above parameters, the RED proportion does not define a concrete progression percentage: different RED proportions are used in different individuals, and always the same proportion is used in one same smile.

### Statistical analysis

The level of agreement between the investigators was assessed based on the Cohen kappa statistic. The Student t-test for dependent samples was used to determine whether the different dimensions of the teeth in the plaster casts and the apparent widths in the photographs exhibited homogeneous values. Testing for independent samples in turn was used to assess sexual dimorphism in the different dimensions and ratios, and a one-sample t-test was used to establish whether a dimension presented a concrete mean value or whether a ratio was adjusted to a certain esthetic proportion. The statistical power for a two-samples Student t-test with a 95% confidence level and considering an effect size of d = 0.3 was 83% for determining gender differences and 99% for determining differences between contralateral teeth. Simple and multiple linear regression models were produced to establish the equation adjusting to the data and predict the width of a missing tooth. In this regard the widths of the LI and CI, and the width of the CI, were regarded as dependent variables, and the widths of the LI and C were regarded as independent variables, in the respective models. Predictive intervals were obtained individually and for the mean, with a 95% confidence level.

## Results

A total of 2304 maxillary anterior tooth (central incisors, lateral incisors and canines) corresponding to 384 subjects were analyzed. There were 178 males (46.4%) and 206 females (53.6%).

**Figure 3:**
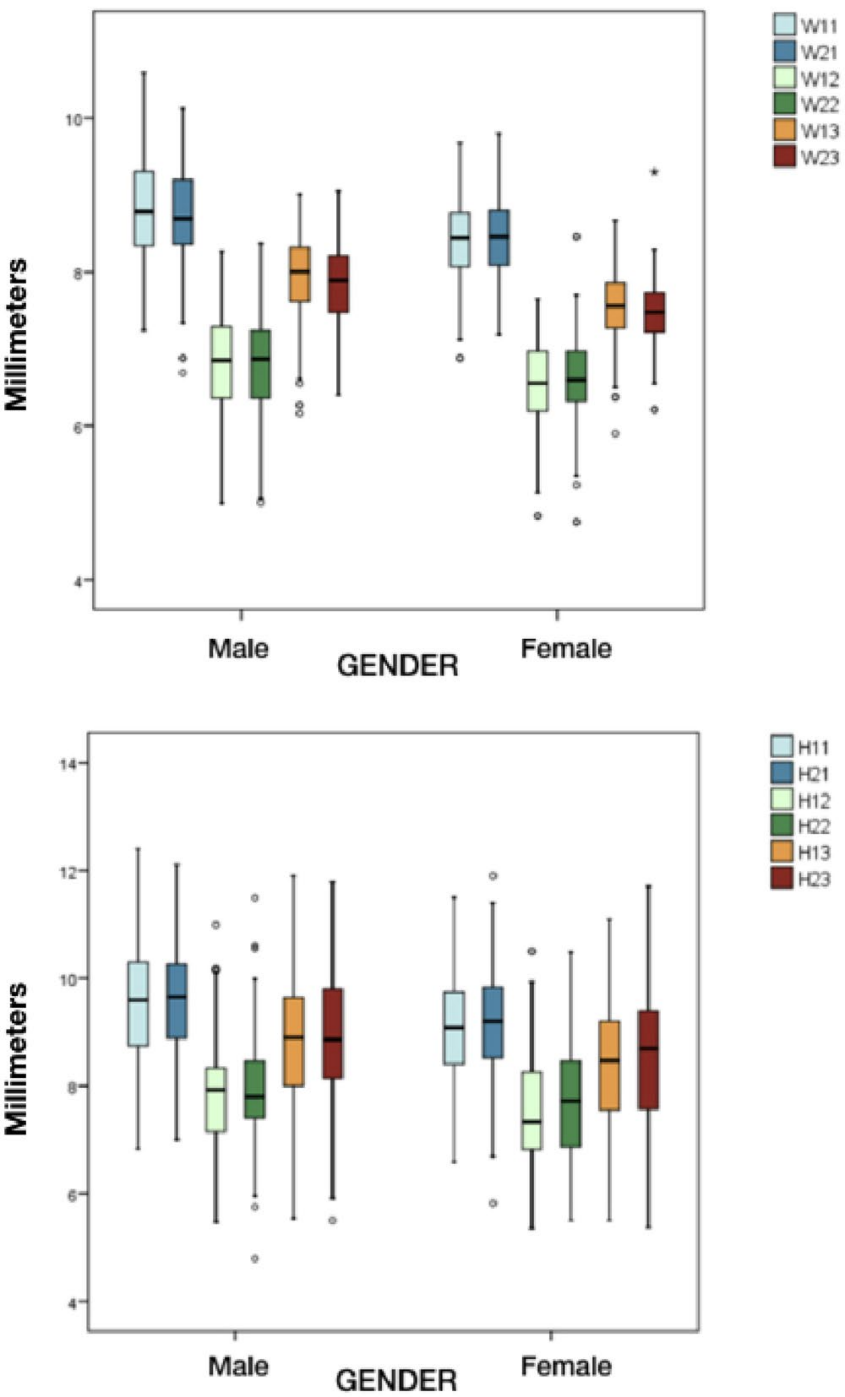
Mean values of height and width of the left and right central incisors, lateral incisors and canines according to gender.

Kappa values for determining inter and intra examiner concordance were 0.84 (p < 0.05) and 0.94 (p < 0.05) respectively, indicating high levels of concordance. Table 1 shows the mean tooth widths according to gender, while Table 2 shows the mean heights. Statistically significant differences were observed on comparing the mean height and width values of the left and right central incisors, lateral incisors and canines according to gender (Fig. 3).

**Table 1:** Distribution of mean width in contralateral teeth and according to gender.

		Gender		
		Total (n = 384)	Males (n = 178)	Females (n = 206)
W 1.1	Mean ± s.d	8.58 ± 0.60	8.79 ± 0.63	8.40 ± 0.51
	Minimum	6.88	7.24	6.88
	Maximum	10.59	10.59	9.68
	Median	8.53	8.79	8.45
W 1.2	Mean ± s.d	6.68 ± 0.63	6.83 ± 0.66	6.54 ± 0.57
	Minimum	4.83	4.99	4.83
	Maximum	8.27	8.27	7.65
	Median	6.69	6.85	6.56
W 1.3	Mean ± s.d	7.73 ± 0.55	7.95 ± 0.57	7.55 ± 0.46
	Minimum	5.90	6.16	5.90
	Maximum	9.01	9.01	8.67
	Median	7.74	8.01	7.56
W 2.1	Mean ± s.d	8.57 ± 0.59	8.73 ± 0.65	8.44 ± 0.51
	Minimum	6.69	6.69	7.19
	Maximum	10.13	10.13	9.80
	Median	8.55	8.70	8.46
W 2.2	Mean ± s.d	6.70 ± 0.64	6.79 ± 0.68	6.62 ± 0.59
	Minimum	4.75	5.00	4.75
	Maximum	8.46	8.37	8.46
	Median	6.71	6.87	6.60
W 2.3	Mean ± s.d	7.65 ± 0.52	7.84 ± 0.53	7.48 ± 0.44
	Minimum	6.21	6.40	6.21
	Maximum	9.30	9.05	9.30
	Median	7.64	7.89	7.48

Note: All measurements were made in millimeters; s.d. = standard deviation.

**Table 2:** Distribution of mean height (H) in contralateral teeth and according to gender.

		Gender		
		Total (n = 384)	Males (n = 178)	Females (n = 206)
H 1.1	Mean ± s.d	9.33 ± 1.01	9.59 ± 1.03	9.10 ± 0.94
	Minimum	6.59	6.83	6.59
	Maximum	12.40	12.40	11.50
	Median	9.25	9.60	9.08
H 1.2	Mean ± s.d	7.67 ± 0.97	7.82 ± 0.95	7.54 ± 0.98
	Minimum	5.35	5.47	5.35
	Maximum	10.99	10.99	10.50
	Median	7.62	7.93	7.33
H 1.3	Mean ± s.d	8.65 ± 1.19	8.91 ± 1.21	8.42 ± 1.12
	Minimum	5.50	5.54	5.50
	Maximum	11.90	11.90	11.09
	Median	8.62	8.90	8.47
H 2.1	Mean ± s.d	9.38 ± 0.98	9.62 ± 0.93	9.18 ± 0.98
	Minimum	5.82	7.00	5.82
	Maximum	12.11	12.11	11.90
	Median	9.38	9.66	9.20
H 2.2	Mean ± s.d	7.82 ± 1.02	7.93 ± 0.98	7.73 ± 1.05
	Minimum	4.79	4.79	5.50
	Maximum	11.49	11.49	10.48
	Median	7.77	7.80	7.72
H 2.3	Mean ± s.d	8.71 ± 1.19	8.93 ± 1.21	8.52 ± 1.14
	Minimum	5.37	5.50	5.37
	Maximum	11.79	11.79	11.71
	Median	8.81	8.86	8.69

Note: All measurements were made in milimeters; s.d. = standard deviation.

The mean width/height ratio in males was 91.9% for the central incisors, 87.2% for the lateral incisors and 89.6% for the canines. Among females the respective ratios were 92.8%, 87.2% and 90%. Sexual dimorphism was confirmed – the difference being statistically significant (p < 0.001). The mean ratios for the global sample were 92.4% for the central incisors, 87.2% for the lateral incisors and 89.8% for the canines. The mean inverse ratios, i.e., height/width, were 1.09 for the central incisors, 1.16 for the lateral incisors and 1.13 for the canines.

Due to the great variability observed among the females, it was not possible to predict the width of the LI knowing the width of the CI (W1 = mean width of the left and right central incisors), or to predict the width of the CI knowing the widths of the LI and C. However, in the case of the males, the width of the LI (W2 = mean width of the left and right lateral incisors) could be predicted knowing the width of the CI, based on the following equation:

$${\rm{W2}}=0.607+0.708\,{\rm{W1}}$$

**Figure 4:**
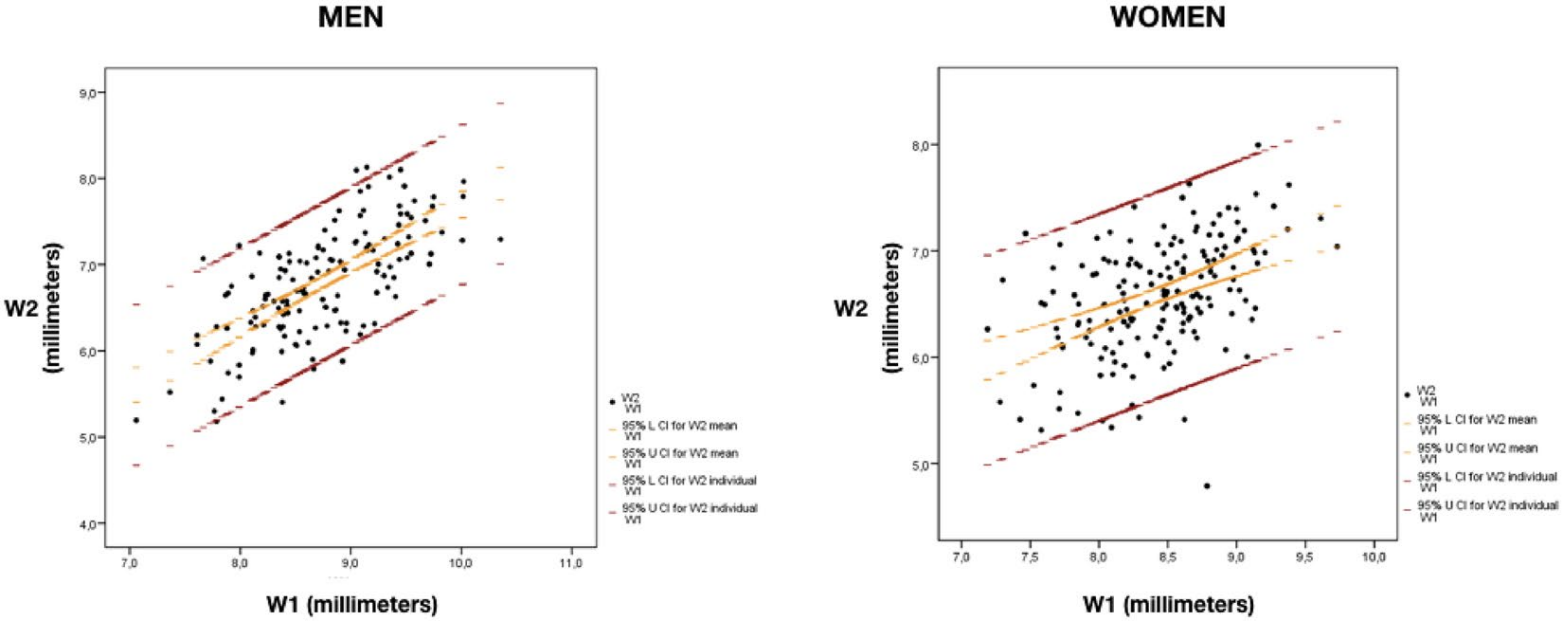
Estimates of both intervals (red for individual and orange for mean) corresponding to parameters W1 (width IC, mean width of the left and right central incisors) and W2 (width IL, mean width of the left and right lateral incisors). L = is the confidence interval lower line. U = is the confidence interval upper line. The figure on the left corresponds to men while the right one is for women. For example, a male with W1 = 9 mm will have a 95% probability of having W2 between 5.9 and 7.8 mm, approximately. However, the mean W2 for all the males with W1 = 9 mm is between 6.8 and 7.0 mm, approximately.

This equation would be able to account for 47.5% of the cases (Fig. 4). In turn, the width of the CI could be predicted in males knowing the widths of the LI and C (W3 = mean width of the left and right canines), based on the following equation:

$${\rm{W1}}=3.301+0.591\,{\rm{W2}}+0.182\,{\rm{W3}}$$

This equation would be able to account for 49.2% of the cases.

Based on the data sample, we take W1, W2, W3 = 0.236, 0.145, 0.119 as the reference values for the apparent teeth dimensions of the average population. On the other hand, we analyzed the anatomy of the gingival margins of the maxillary anterior teeth, determining whether the relationships between them were positive or negative. In 81.8% of the sample, the margin of the right lateral incisor was negative, i.e., it was located below the margins of the central incisor and canine, while in 18.2% of the cases the margin was positive or null. Furthermore, the proportion of negative cases was significantly higher in males (88.2%) than in females (76.2%) (p = 0.002). In 88.3% of the sample, the margin of the left lateral incisor was negative, while in 11.7% of the cases the margin was positive or null. There were no gender differences (p = 0.363).

**Figure 5:**
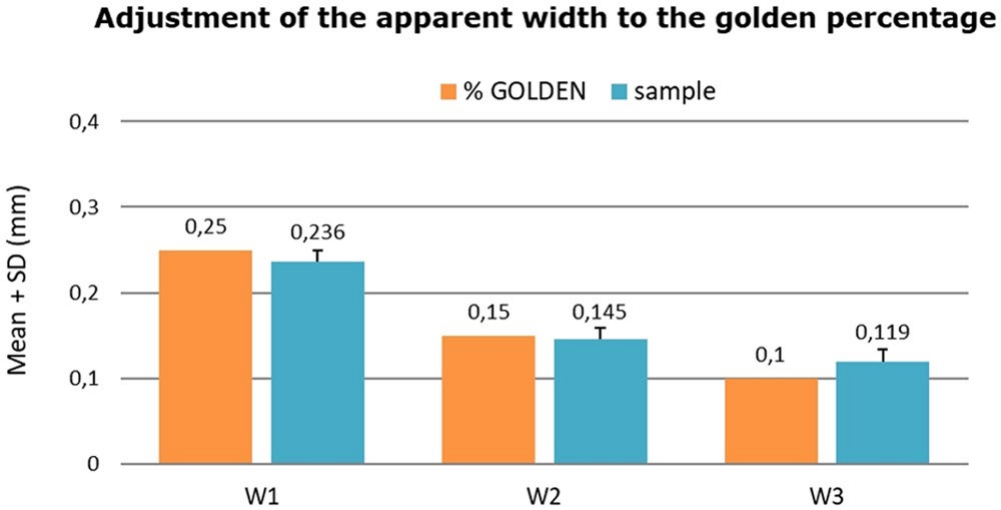
Adjustment of apparent widths of the central incisor, lateral incisor and canine to the Golden percentage.

In order to determine whether the Golden proportion was followed, we calculated the LI/CI ratio, which yielded a mean value of 0.61 (p = 0.648), versus 0.83 in the case of the C/LI ratio (p < 0.001). The Golden percentage in turn was not followed by any of the analyzed teeth. The apparent widths in relation to intercanine distance complied with the proportions of the Golden percentage (p < 0.001). The values obtained were close to the reference values (0.236, 0.145 and 0.119), but in statistical terms were significantly different (Fig. 5).

On examining the apparent widths in relation to the RED proportion, the mean value was found to be 0.778 (p < 0.001). The proportion of the lateral incisor with respect to the central incisor was smaller (22%) than the proportion of the canine with respect to the lateral incisor. None of the teeth in the sample followed the RED proportion.

## Discussion

The results of this analytical observational study describe the dimensions of 2304 maxillary anterior tooth in 384 subjects. In addition, the relationships of these dimensions among the central and lateral incisors and canines were explored with a view to using them during rehabilitation of the maxillary anterior sector. The Golden proportion, Golden percentage and RED proportion were not followed by most of the subjects in the study. Consequently, while these “standards” have been classically used as a guide in dental esthetics, they were not found to be clinically useful in our series.

Reducing smile subjectiveness is an important concern. It is therefore clinically relevant to determine the relationships among the dimensions of the teeth in order to secure esthetic and reproducible outcomes^1,2^. The different studies published to date are characterized by a lack of homogeneity in the analytical methods used. Some authors perform measurements on anatomical crowns of extracted teeth^14,15^, while others obtain digital photographs to measure the clinical crown using virtual calipers^16,17^. Plaster casts have also been used^18,19^. These methodological differences make it difficult to compare the different published studies. Variability is moreover also observed in the methods used to determine the proportions. Some studies use the Levin grid^17,18^, while others base their calculations on digital photographs^19,20^. It therefore would be of great interest to define a standardized protocol allowing the systematic conduction of studies, with adequate comparisons of the results obtained.

In most cases it is assumed that the matching teeth on both sides have similar or even identical mesiodistal and gingivo-incisal diameters. This is not always the case, however. Marvouskoufis *et al*.^21^ found that the central incisors did not have exactly the same dimensions in 86–90% of the studied subjects. In contrast, other studies^19,22^ have reported no significant differences in mean dimensions with respect to the contralateral teeth. In our series there were statistically significant differences of between 0.05–0.018 mm referred to both width and height. Such differences might not be, however, of clinical relevance.

**Figure 6:**
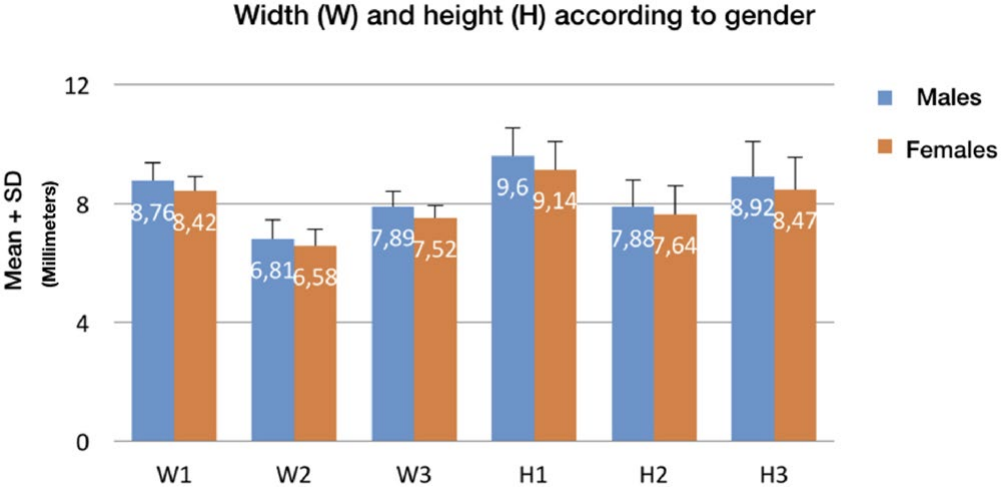
Mean widths and heights of the central incisor, lateral incisor and canine according to gender.

On the other hand, sexual dimorphism referred to the dimensions of teeth has also been the subject of debate. Some authors have reported no statistically significant dimorphism in the width and height of the maxillary anterior teeth^12,23^, while others have indeed observed gender differences^16,24^. Those studies that have reported sexual dimorphism describe greater dental width and height in males compared with females^11,19,24^. This is consistent with the results obtained in our series, where males presented a mean height 0.46-0.24 mm greater than in females, depending on the type of tooth. The width values in turn were 0.37-0.23 mm greater in males (p < 0.001) (Fig. 6).

The literature describes great variability in percentage width/height ratio. According to Brisman^25^, a width/height ratio of 75% is to be preferred, in coincidence with Magne *et al*.^14^ for the lateral incisors (78%) and canines (73%). Other investigators have concluded that the maxillary anterior teeth should have a ratio of about 80% in order to ensure optimum esthetic effects, particularly in the case of the central incisors^10,26^. Nevertheless, other authors advocate ratios of over 80%^12,19,27^. Orozco-Varo *et al*.^28^ described a mean width/height ratio of 85% for the central incisors, with a decrease in ratio to 79% in the case of the lateral incisors and canines. Hasanreisoglu *et al*.^11^ in turn reported a range of 72–124%, and found higher width/height ratios to correspond to square teeth, i.e., shorter and/or wider teeth^11,12^, in coincidence with the observations of Rossentiel *et al*.^10^. The review of the studies published to date yielded no percentages similar to the Golden proportion (62%) for the width/height ratio of the maxillary incisors. Indeed, the reported percentage ratios were seen to be higher^12,14,25^, in the same way as in our study. In the present series the mean ratio was 92.4% for the central incisors, 87.2% for the lateral incisors and 89.8% for the canines. Similar results have been obtained in studies involving German Caucasian, Nepalese and North American populations^6,17,26^.

The reference height/width ratio has been less widely studied, and in most cases the abovementioned width/height ratio is used. The values in our series were lower than those reported by other investigators^11,14,19^, who have described proportions of between 1.11^11^ and 1.28^14^ for the central incisors, a maximum ratio of between 1.37^14^ and 1.18^19^, and ratios of between 1.37^14^ and 1.17 for the canines^11,19^.

The most significant parameter in relation to the soft tissues is the margin gingival. In the present study, 81.8% of the upper right incisors had a gingival margin below or at the same height as the margin of the central incisors and canines. This finding was in concurrence with other published studies^13,29,30^. Chu *et al*.^13^ found only 15% of lateral incisor’s gingival margin at the same level of central incisors and canines. Furthermore, in this study, the proportion of negative values was significantly higher in males (82.2%) than in females (76.2%). In the case of the upper left incisors the figure was slightly higher (88.3%) and no gender differences were noted. This result is in agreement with other reported by Charruel *et al*.^29^ who showed an asymmetry between left-right side, indicating that perfect body symmetry does not exist. Kolte *et al*.^30^ found a difference (p > 0.05) between males and females, where the distance between gingival margin of lateral incisor to the level of canine and central incisor is higher in females. It could be explained because of the predominant triangular crown shape that women have, which is associated with thin gingival biotype. The distance from the gingival marginal of the lateral incisor in relation to the line joining the tangent of the margin of the central incisor and canine was 1 ± 0.2 mm.

Several studies have documented recurrent relationships among the apparent widths of the anterior teeth seen in frontal view. Levin^7^ used the Golden proportion. Some investigators^31^ advocate this proportion as esthetically pleasant. Nevertheless, this is not the predominant opinion^11,32,33^: if the Golden proportion were followed, the dental arch would be very narrow, the canines seen in frontal view would be reduced in width, and the central incisors would be more prominent^19,34^. In our study we obtained values of 0.616 for the LI/CI apparent width ratio and 0.832 for the C/LI ratio – these figures being consistent with those recorded in other studies^17,32,34^. The mean LI/CI ratio was practically coincident with the Golden proportion, while the C/LI ratio differed – the canines being markedly wider than contemplated by the Golden proportion. Other authors^19^ have described LI/CI and C/LI ratios that differ from this proportion. Preston^33^ recorded the Golden proportion in only 17% of the cases, while other investigators^20,23^ have documented higher percentages of 34.9% and 38.2%, though even so statistical significance was not reached.

Some studies have observed no correlation to the Golden proportion but nevertheless have reported recurrent proportions between CI/LI and C/LI^10,35^. Ward *et al*.^6^ were the first to consider the importance of the height of the teeth in determining the width of the maxillary anterior teeth. In the case of central incisors of a considerable height, scant width of the lateral incisor and canine would be desirable for an esthetic smile. The RED proportion therefore would present low values, while in the case of short teeth a greater width of the lateral incisor and canine would yield a more esthetic effect – with a correspondingly higher RED proportion^6^. In our study we observed no repeated or recurrent proportion, in concordance with the findings of most studies^11,36^.

With regard to the Golden percentage for correlating the teeth of the anterior sector, the results of our study found the mean value of the central incisors to be 23.6% - this being practically identical to the figures obtained in other studies^32,37^ and lower than the value suggested by the Golden percentage^8^. The mean value of the Golden percentage for the lateral incisors was 14.5%, in coincidence with the study of Ali Fayyad *et al*.^32^. This figure can be considered equivalent to that originally defined by Snow. With regard to the percentage in the case of the canines, the results of our study indicated a mean value of 11.9%, while other authors have recorded slightly higher values of up to 13%^37^. Although the differences are statistically significant, they may go unnoticed from the clinical perspective.

The main limitation of our study is referred to the dental casts used, since we first obtained imprints with irreversible hydrocolloid material, followed by plaster casting, and this may have introduced minor discrepancies. Nevertheless, it must be mentioned that the study sample is one of the largest reported to date, involving over 380 subjects and with a statistical power of 99%. Furthermore, we have presented several formulas allowing us to predict the width of a missing maxillary anterior tooth from the existing teeth, in the male population.

## Conclusions

A total of 2304 teeth corresponding to 384 subjects were revisited, and in this study were not seen to comply with the ideal classical aesthetic dimensions in the anterior maxillary sector. The Golden proportion, Golden percentage and RED proportion were not found between the mesiodistal width of the maxillary central incisors and lateral incisors, or between mesiodistal width of the maxillary lateral incisors and canines. The information obtained from this study could be used as reference during oral rehabilitation of the esthetically sensitive anterior sector and may be clinically applied to restore the dimensions during periodontal surgery, restorative dentistry and prosthetic rehabilitation.
